# A relationship between attractiveness and performance in professional cyclists

**DOI:** 10.1098/rsbl.2013.0966

**Published:** 2014-02

**Authors:** Erik Postma

**Affiliations:** Institute of Evolutionary Biology and Environmental Studies, University of Zurich, Winterthurerstrasse 190, 8057 Zürich, Switzerland

**Keywords:** endurance performance, cycling, humans, mate preference, sexual selection

## Abstract

Females often prefer to mate with high quality males, and one aspect of quality is physical performance. Although a preference for physically fitter males is therefore predicted, the relationship between attractiveness and performance has rarely been quantified. Here, I test for such a relationship in humans and ask whether variation in (endurance) performance is associated with variation in facial attractiveness within elite professional cyclists that finished the 2012 Tour de France. I show that riders that performed better were more attractive, and that this preference was strongest in women not using a hormonal contraceptive. Thereby, I show that, within this preselected but relatively homogeneous sample of the male population, facial attractiveness signals endurance performance. Provided that there is a relationship between performance-mediated attractiveness and reproductive success, this suggests that human endurance capacity has been subject to sexual selection in our evolutionary past.

## 1. Introduction

Choosy females prefer to mate with high quality males, because they make ‘good fathers’ (direct benefits), and/or because they provide ‘good genes’ for their offspring (indirect benefits) [1]. One aspect of quality is whole-organism performance, defined as any quantitative measure of how well an organism performs an ecologically relevant, dynamic behaviour [2]. In non-human animals, for example, locomotor performance is often positively associated with fitness [3]. However, whereas the importance of performance in shaping the outcome of male–male interactions has been shown repeatedly, less is known about its importance in the context of female mate choice [2].

In humans, the link between attractiveness and quality has proved elusive [4], and the few studies that have quantified the link between attractiveness and performance typically used a random sample from the general population ([5], but see [6]). In such a sample, there are many variables that affect attractiveness and/or performance, including differences in training and diet, which may obscure or generate associations between the two. Also, the measures of performance employed predominantly capture variation in strength and coordination, rather than endurance, which is more difficult to quantify. However, it has been hypothesized that it is endurance capacity in particular, that has been subject to strong selection in our evolutionary past [7,8].

Here, I use data from elite professional cyclists that finished the 2012 Tour de France, generally considered to be one of the hardest endurance events. In this unique subset of the male population, which is relatively homogeneous in terms of training effort and motivation, I test for a relationship between attractiveness and performance. Furthermore, I test whether this relationship is stronger when attractiveness is scored by naturally cycling women as compared with when scoring is done by women using a hormonal contraceptive or men [9].

## 2. Material and methods

### (a) Measuring attractiveness

Eighty portraits of riders that participated in the 2012 Tour de France, taken on the day before the start of the race, were obtained from http://www.letour.fr, together with their date of birth, nationality, height and weight. Portraits showed the head, neck and part of the shoulders and were standardized in terms of lighting, distance and background.

I created two online surveys, each containing the portraits of 40 riders in a random order, at http://www.fluidsurveys.com. Participants were first asked to rate each rider in terms of attractiveness on a discrete scale from 1 to 5, with 5 being the highest. Before moving on to the next portrait, raters were asked to also provide a masculinity and a likeability score for this rider. Masculinity may be correlated with attractiveness [10] and mediate a relationship between attractiveness and performance, and likeability captures variation in facial expression (i.e. smiling).

In addition, participants provided information on, among other things, their sex and age, and on whether they thought they knew the rider. Furthermore, women were asked whether they used a hormonal contraceptive, and if they did not, for the average length of their cycle and how many days had passed since the start of their last period.

In total, 398 + 418 = 816 people participated, 72% of which were female (for more demographic information, see the electronic supplementary material, Results). A total of 282 out of a total 32 468 attractiveness ratings (0.9%) were excluded because the rater indicated that he or she recognized the rider. For more information on rider selection and data collection, the inference of female fertile phase, variation in facial expression of the riders, and on rider height and weight, see the electronic supplementary material, Methods.

### (b) Quantifying performance

To quantify rider performance, I performed a principal component analysis on the time it took for each rider to complete the prologue, the two individual time trials and the complete race (minus the time for the prologue and the time trials). I extracted the first principal component, and to ensure faster riders had higher values, multiplied this with −1 (for details, see the electronic supplementary material, Methods).

### (c) Statistical analyses

I used linear mixed models using restricted maximum likelihood (REML) to test for systematic differences in attractiveness among riders and raters by fitting rider and rater identity as random effects, and assessed their significance using one-sided likelihood-ratio (LR) tests.

I subsequently tested whether performance was a predictor of attractiveness by including performance, as well as various rater-specific variables that might explain additional variation in attractiveness scores. Note that at this stage, no other rider-specific variables (e.g. age or weight) were included, as these might be mediators of a relationship between attractiveness and performance. For all covariates, both linear and quadratic terms were fitted. Rater nationality was fitted as a random effect. I performed backward elimination of non-significant terms, starting with the least significant quadratic terms. Significance of fixed effects was assessed using LR tests (using maximum likelihood (ML)). Parameter estimates of significant terms were obtained from the final model (fitted using REML), and for non-significant terms they were obtained by reintroducing them one-by-one into the final model.

Having estimated the overall effect of performance on attractiveness, other rider-specific variables were included into the model arrived at above, again followed by backward elimination. Note that starting with a full model including all rider- and rater-specific variables resulted in the same final model. The proportion of variance in attractiveness among riders and raters explained by the rider- and rater-specific fixed effects retained in the final model was calculated following [11].

Finally, I tested for rater-specific variation in the relationship between attractiveness and performance by expanding the model arrived at above with a random slope for the regression of attractiveness on performance for each rater, and included an interaction between performance and various rater-specific variables. Note that whereas the effect of performance on attractiveness is tested on the level of the rider (*N* = 80), interactions between performance and rater-specific variables are tested on the level of the rater (*N* = 816).

I repeated all analyses for masculinity and likeability, as well as for attractiveness corrected for likeability and vice versa. Residual attractiveness, masculinity and likeability scores were normally distributed. All analyses were run in R v. 3.0.0 [12]. Linear mixed models were run using lme4 0.999999-2 [13].

## 3. Results

### (a) Variation in attractiveness

**Figure 1.**
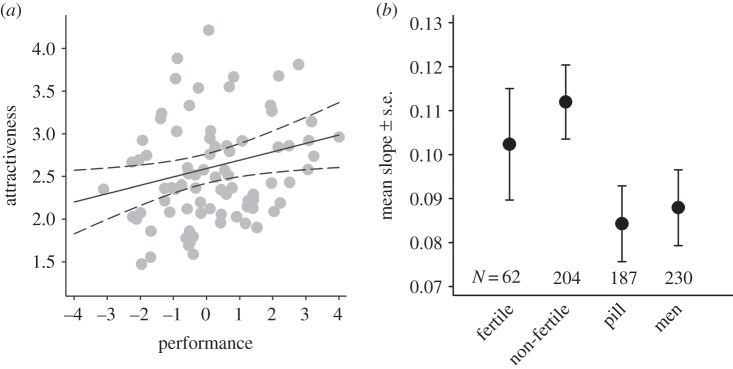
(*a*) The relationship between attractiveness and performance. Grey dots depict a rider's attractiveness score, averaged across raters and plotted against his performance. The solid and dashed lines depict the relationship between attractiveness and performance and its 95% CI, obtained from a mixed model including additional rider- and rater-specific variables. (*b*) The mean rater-specific slope of this relationship and its standard error, for women in the fertile part of their cycle, women in the non-fertile part of their cycle, pill-using women and men. Also see the electronic supplementary material, figure S1.

There is significant variation among riders in attractiveness, with rider ID explaining 28% of the variation in attractiveness scores (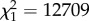, *p* < 0.001). Part of this variation is associated with their performance during the 2012 Tour de France, with better performing riders receiving on average higher attractiveness scores (*b* = 0.091 ± 0.043, 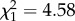, *p* = 0.032, *R*^2^ = 5.5%; figure 1*a*; electronic supplementary material, figure S1). In those riders that also took part in the 2013 Tour de France, there is a very similar association with their performance in that year (see electronic supplementary material, Results).

Including additional rider-specific variables showed a quadratic effect of rider age (age^2^: *b* = −0.0064 ± 0.0033, 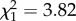, *p* = 0.051; *R*^2^ = 4.8%), with riders aged 29.6 being most attractive. Furthermore, taller and heavier riders were rated as more attractive (*b* = 0.13 ± 0.052, 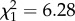, *p* = 0.012, *R*^2^ = 7.0%), but there was no effect of relative weight (*b* = 0.017 ± 0.12, 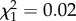, *p* = 0.89; see electronic supplementary material, Methods). Rider nationality explained no variation in attractiveness. Also, there was no effect of facial expression on attractiveness (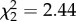, *p* = 0.30; electronic supplementary material, figure S2). Importantly, including rider size and age did not affect the relationship between performance and attractiveness (*b* = 0.098 ± 0.043, 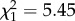, *p* = 0.020, *R*^2^ = 5.9%; figure 1*a*).

Which rider-specific variables shape performance, and which rater-specific variables shape attractiveness scores, is outlined in the electronic supplementary material, Results.

### (b) Variation in the relationship between performance and attractiveness

Despite substantial individual variation (see electronic supplementary material, figure S1), the slope of attractiveness on performance differed significantly among female raters using the pill, female raters in the non-fertile part of their cycle, female raters in the fertile part of their cycle and male raters (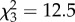, *p* = 0.006; figure 1*b*; electronic supplementary material, figure S1c). Although still positive, the slope was significantly weaker in men and pill-using women (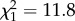, *p* < 0.001). There was no significant difference in the slope between men and pill-using women (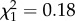, *p* = 0.67) or between women in the fertile and in the non-fertile part of their cycle (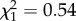, *p* = 0.46). None of the interactions between other rater-specific variable and performance were significant (see electronic supplementary material, Results).

### (c) Masculinity and likeability

There was no association between performance and masculinity, whereas there was a positive association between performance and likeability. The significant relationship between attractiveness and performance, as well as the significant difference between men and pill-using women versus non-pill-using women remained when attractiveness was corrected for likeability, whereas there was no relationship between performance and likeability corrected for attractiveness (see electronic supplementary material, Results).

## 4. Discussion

Why is there an association between a rider's attractiveness and his performance during the Tour de France? First, performance may be positively correlated with general health, vigour or strength, or certain personality characteristics (e.g. competitiveness), which in their turn may be associated with attractiveness. Alternatively, facial attractiveness may signal endurance performance in particular. Indeed, high endurance performance is thought to have been the target of selection in early hominids, as being able to efficiently cover large distances allowed for more efficient hunting, gathering and scavenging, resulting in a number of uniquely human adaptations [7].

If true, individuals with higher endurance capacity were likely to be better resource providers for their partner and progeny. By choosing a mate with high endurance capacity, a woman would thus have gained direct (e.g. more resources for her and her offspring) and/or indirect (i.e. physically fitter offspring) benefits. Interestingly, across cultures, women place a lot of value on the provisioning ability of their prospective partner [14]. So, provided the association of endurance performance (i.e. physical fitness) with attractiveness translates into an association with reproductive success (i.e. evolutionary fitness) [15], endurance performance may have been subject to natural as well as sexual selection [8].

Although their preference was significantly weaker, also (heterosexual) men rated faster cyclists as more attractive. Furthermore, there was a close correlation between male and female ratings (see electronic supplementary material, Results). This suggests that men either know what (heterosexual) women find attractive, or that preference functions for performance-mediated attractiveness are to some degree independent of sex. Also pill-using women showed a reduced preference for faster cyclists. Although the difference is relatively small and women using the pill are not a random subset of the female population, this is in line with other studies demonstrating a reduced preference for indicators of male quality in pill-using women [9].

To summarize, I was able to simultaneously investigate the effects of several rider- and rater-specific variables on attractiveness scores and show a relationship between facial attractiveness and performance. Although the mechanism mediating this relationship remains to be elucidated, this provides a fascinating new insight into the nature of human endurance performance.

## Data accessibility

Data available from the Dryad Digital Repository: http://doi.org/10.5061/dryad.v77j2.

## References

[1] Andersson M 1994 Sexual selection. Princeton, NJ: Princeton University Press

[2] Lailvaux SP Irschick DJ 2006 A functional perspective on sexual selection: insights and future prospects. Anim. Behav. 72, 263–273 (doi:10.1016/j.anbehav.2006.02.003)

[3] Irschick D Bailey JK Schweitzer JA Husak JF Meyers JJ 2007 New directions for studying selection in nature: studies of performance and communities. Physiol. Biochem. Zool. 80, 557–567 (doi:10.1086/521203) 17909993 10.1086/521203

[4] Scott IML Clark AP Boothroyd LG Penton-Voak IS 2013 Do men's faces really signal heritable immunocompetence? Behav. Ecol. 24, 579–589 (doi:10.1093/beheco/ars092) 23555177 10.1093/beheco/ars092 PMC3613940

[5] Hönekopp J Rudolph U Beier L Liebert A Muller C 2007 Physical attractiveness of face and body as indicators of physical fitness in men. Evol. Hum. Behav. 28, 106–111 (doi:10.1016/j.evolhumbehav.2006.09.001)

[6] Williams KM Park JH Wieling MB 2010 The face reveals athletic flair: Better National Football League quarterbacks are better looking. Pers. Individ. Differ. 48, 112–116 (doi:10.1016/j.paid.2009.09.003)

[7] Bramble DM Lieberman DE 2004 Endurance running and the evolution of *Homo*. Nature 432, 345–352 (doi:10.1038/nature03052) 15549097 10.1038/nature03052

[8] Heinrich B 2001 Why we run: a natural history. New York, NY: HarperCollins Publishers Inc

[9] Alvergne A Lummaa V 2009 Does the contraceptive pill alter mate choice in humans? Trends Ecol. Evol. 25, 171–179 (doi:10.1016/j.tree.2009.08.003) 19818527 10.1016/j.tree.2009.08.003

[10] Rhodes G 2006 The evolutionary psychology of facial beauty. Annu. Rev. Psychol. 57, 199–226 (doi:10.1146/annurev.psych.57.102904.190208) 16318594 10.1146/annurev.psych.57.102904.190208

[11] Nakagawa S Schielzeth H 2013 A general and simple method for obtaining R2 from generalized linear mixed-effects models. Methods Ecol. Evol. 4, 133–142 (doi:10.1111/j.2041-210x.2012.00261.x)

[12] R Core Team 2013 R: a language and environment for statistical computing, version 3.0.0. Vienna, Austria: R Foundation for Statistical Computing

[13] Bates DM Maechler M Bolker B 2013 lme4: linear mixed-effects models using S4 classes. R package version 0.999375–31 See http://CRAN.R-project.org/package=lme4

[14] Buss DM 1989 Sex-differences in human mate preferences: evolutionary hypothesis tested in 37 cultures. Behav. Brain Sci. 12, 1–14 (doi:10.1017/S0140525X00023992)

[15] Jokela M 2009 Physical attractiveness and reproductive success in humans: evidence from the late 20th century United States. Evol. Hum. Behav. 30, 342–350 (doi:10.1016/j.evolhumbehav.2009.03.006) 21151758 10.1016/j.evolhumbehav.2009.03.006 PMC3000557

